# Exercise Hemodynamics and Sex-Specific Data in Asymptomatic Adults: An Exploratory Pilot Study

**DOI:** 10.3390/diagnostics15111307

**Published:** 2025-05-23

**Authors:** Mi-Hyang Jung, So-Young Lee, Woo-Baek Chung, Jong-Chan Youn, Hae Ok Jung

**Affiliations:** 1 Division of Cardiology, Department of Internal Medicine, Seoul St. Mary’s Hospital, College of Medicine, The Catholic University of Korea, Seoul 06591, Republic of Korea; floria0515@gmail.com (M.-H.J.); sarahsylee777@gmail.com (S.-Y.L.); peace816@catholic.ac.kr (W.-B.C.); jong.chan.youn@gmail.com (J.-C.Y.); 2 Catholic Research Institute for Intractable Cardiovascular Disease, College of Medicine, The Catholic University of Korea, Seoul 06591, Republic of Korea

**Keywords:** exercise test, exercise tolerance, hemodynamics, sex

## Abstract

**Background**: Understanding normal exercise hemodynamics is essential for assessing individuals with exertional dyspnea. This study utilized exercise echocardiography to gain insights into exercise hemodynamics in asymptomatic middle-aged to older adults without overt cardiovascular disease. **Methods**: We prospectively enrolled 30 individuals aged 45–75 years without dyspnea, excluding those with left ventricular ejection fraction (LVEF) < 50% or significant heart/lung diseases. All participants underwent symptom-limited bicycle exercise echocardiography. **Results**: Two individuals exhibited early-stage dyspnea, leading to the inclusion of 28 individuals (mean age 61 ± 8 years, 50% female) in the final analysis. Throughout the exercise, the average E/e’ ratio increased from 8.3 ± 1.6 at rest to 9.7 ± 1.8 at 75 W (*p* = 0.001), while systolic pulmonary artery pressure (SPAP) rose from 23.0 ± 3.9 mmHg at rest to 41.2 ± 9.3 mmHg at 75 W (*p* < 0.001). Sex-specific analysis revealed a more pronounced elevation in SPAP during exercise among females (SPAP at 75 W, 45.5 ± 8.3 in females; 36.8 ± 8.3 mmHg in males, *p* = 0.011; *p* < 0.001 for interaction between sexes). **Conclusions**: In asymptomatic middle-aged to older adults, while there was a slight increase in left ventricular filling pressure and SPAP during exercise, the mean average E/e’ and SPAP at peak exercise were below 10 and 50 mmHg, respectively. Our findings also demonstrate sex-specific differences, with females exhibiting a more pronounced elevation in SPAP during exercise.

## 1. Introduction

With an aging population and an increase in cardiometabolic comorbidities, the prevalence of heart failure with preserved ejection fraction (HFpEF) is gradually increasing [1,2]. Additionally, the recent introduction of an effective medication for HFpEF, such as the sodium-glucose cotransporter 2 inhibitor, has facilitated a higher rate of HFpEF diagnosis because more clinicians actively identify patients with this condition [3,4]. When the elevation of natriuretic peptide is combined with echocardiographic evidence of cardiac structure and hemodynamic abnormality supporting elevated left ventricular filling pressure (LVFP), the diagnosis of HFpEF could be established. Unfortunately, some patients do not meet the pre-specified cutoff value for resting E/e’ (a representative marker of LVFP), necessitating further examinations, such as exercise echocardiography or cardiac catheterization [5,6]. Therefore, recognizing normal exercise hemodynamic values and patterns becomes crucial for identifying abnormal exercise hemodynamic profiles.

Bicycle exercise echocardiography is a noninvasive modality that enables a confirmatory diagnosis of HFpEF [5,6,7,8,9,10]. Unlike cardiac catheterization, it can be performed in an outpatient clinic with minimal side effects, enhancing its applicability in a large population. In contrast to treadmill exercise echocardiography, it can track the entire exercise stage without interruption, facilitating the identification of exercise kinetics [10,11,12]. Furthermore, it is more accessible for individuals with arthralgia to undergo the test. Previous studies utilizing exercise echocardiography have limitations, either related to the diagnostic modality (typically a treadmill test that solely evaluates resting and peak exercise hemodynamics immediately after exercise [13]) or the study population (often comprising young adults [14,15] or symptomatic individuals [16]).

In this context, we conducted the current study to understand the exercise hemodynamics among individuals aged 45–75 years without dyspnea using bicycle exercise echocardiography. Additionally, we aimed to explore sex-specific differences in this population.

## 2. Methods

### 2.1. Study Design and Study Population

**Figure 1:**
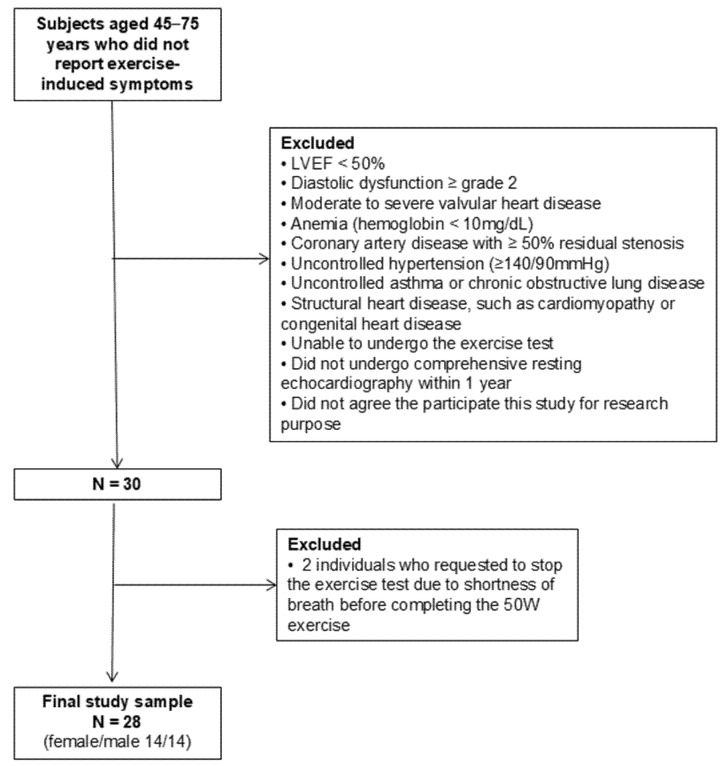
Flowchart of the study population. Abbreviations: LVEF, left ventricular ejection fraction.

This is a prospective observational study with cross-sectional analysis. We prospectively and consecutively enrolled 30 individuals aged 45–75 years, who did not report exercise-related symptoms and agreed to participate in the present study, between June 2022 and July 2023 at a single tertiary hospital. Exclusion criteria included individuals with left ventricular ejection fraction (LVEF) < 50%, diastolic dysfunction ≥ grade II at resting echocardiography [9], significant (moderate or severe) valvular heart disease, anemia (hemoglobin < 10 mg/dL), coronary artery disease with ≥50% residual stenosis, uncontrolled hypertension (≥140/90 mmHg), uncontrolled asthma or chronic obstructive lung disease, and structural heart diseases such as cardiomyopathy or congenital heart disease. We also excluded individuals with arthralgia who were unable to undergo the exercise test. Only those who had undergone comprehensive resting echocardiography within the last year, confirming their eligibility against the inclusion and exclusion criteria, were included. All participants consented to engage in bicycle exercise echocardiography for research purposes. Among the 30 individuals, two participants who requested to stop the exercise test due to shortness of breath before completing the 50 W exercise were excluded from the final study cohort, resulting in a total of 28 individuals included in the analysis. The flow chart of the study population enrollment is summarized in Figure 1. The local institutional review board granted approval for the study protocol (KC22OISI0384), and written informed consent was obtained from all participants. All procedures were conducted in accordance with the ethical standards of the institutional research committee and with the 1964 Declaration of Helsinki and its later amendments or comparable ethical standards.

### 2.2. Bicycle Exercise Echocardiography

During bicycle exercise echocardiography, individuals engaged in exercise on a specialized bicycle (eBike EL tilt-table ergometer; GE HealthCare-Ergoline GmbH, Bitz, Germany) positioned in a semi-supine posture. Simultaneously, a Doppler echocardiographic examination using a commercially available system (Vivid E95; GE HealthCare, Horten, Norway) was conducted at each exercise stage to monitor heart function. The exercise protocol involved increasing intensity every 3 min, starting at 25 W and rising incrementally by 25 W until reaching the target heart rate, achieving a maximum workload of 100 W, or experiencing exercise-limited symptoms [7]. Echocardiographic parameters were analyzed at each stage up to 75 W, which all participants completed successfully. Although the protocol permitted exercise up to 100 W, diastolic parameters were not assessed beyond 75 W because the heart rates at this stage typically exceeded 110 bpm, frequently resulting in E/A wave summation and limiting the reliability of diastolic measurements. Importantly, participants who could perform at 100 W or more did not exhibit a significant increase in tricuspid regurgitation (TR) velocity during the entire exercise session. In cases where exercise intensity exceeded 75 W, we analyzed the total exercise time. The participants were directed to maintain a pedaling speed of 60 rotations per minute (rpm), with an allowable range between 55 and 65 rpm. At each exercise stage, blood pressure, heart rate, oxygen saturation, and electrocardiography were monitored along with echocardiographic data. For echocardiographic data, 2D cine loops of three cardiac cycles were acquired in the apical four chamber view and right ventricle (RV) focused view. After termination of exercise, recovery phase images were acquired at 2 min and 5 min, respectively.

### 2.3. Doppler Echocardiographic Measurements During Resting and Exercise

Systolic and diastolic function parameters were digitally recorded at rest, during exercise, and post-exercise, and analyzed upon study completion. Diastolic parameters included mitral inflow velocity (E and A wave velocity), mitral annular velocity (septal and lateral e’ velocity), E/e’ ratio (septal and average E/e’ value), TR velocity, estimated systolic pulmonary artery pressure (SPAP), left atrial (LA) volume index, and LA reservoir strain [17]. SPAP assumed a right atrial pressure (RAP) of 5 mmHg. Systolic parameters included LVEF and left ventricular (LV) global longitudinal strain. During exercise, LVEF and LV global longitudinal strain were evaluated in a single plane from the apical four-chamber view. As ancillary variables, parameters for RV function (RV free wall strain and tricuspid annular plane systolic excursion [TAPSE]) and RV–pulmonary artery (PA) coupling index (TAPSE/SPAP ratio, expressed in mm/mmHg) were tracked to explore dynamic changes during exercise [18,19]. LA strain (biplane) was only evaluated at rest; otherwise, all parameters were repeatedly measured at each exercise stage. Among these various parameters, E/e’ ratio and SPAP were the main parameters reflecting diastolic function.

### 2.4. Statistical Methods

The continuous variables were presented as mean ± standard deviation, and categorical variables were presented as numbers with percentages. The normality of data distribution was assessed using the Shapiro–Wilk test, which confirmed that all key variables followed a normal distribution. A linear mixed-effects model was employed to assess dynamic changes during exercise, and post-hoc analysis with Bonferroni correction was conducted. A Student’s *t*-test was used for continuous variables in analyzing the sex-specific hemodynamic data, and the Chi-square test or Fisher’s exact test was applied for categorical variables, depending on cell counts. To examine exercise-induced changes (Δ), the difference between the resting and peak exercise parameters was calculated. To verify the consistency of the exercise echocardiographic parameters, both inter- and intra-observer agreement were assessed by computing the intraclass correlation coefficient (ICC). An ICC exceeding 0.8 was considered to indicate excellent agreement. A *p*-value of <0.05 was considered statistically significant. All analyses were performed using SPSS version 21.0 software (IBM).

## 3. Results

### 3.1. Baseline Characteristics

A total of 28 individuals (14 females and 14 males) were included in the final analyses. As previously mentioned, two individuals (6.7%) were excluded from the current analyses due to exercise-limiting dyspnea that developed early in the exercise, even though they did not report any symptoms before participating in the test. The mean age was 60.9 ± 8.4 years, with 60.7% falling within the age range of 60–74, and the mean body mass index was 24.6 ± 4.0 kg/m^2^. All participants had sinus rhythm. Among them, 60.7% had hypertension, and 25.0% had diabetes. The mean systolic blood pressure of all participants was 119 ± 8 mmHg, and among those with diabetes, the mean HbA1C was 6.6 ± 0.4%. The resting LVEF (62.9 ± 2.3%), average E/e′ ratio (8.3 ± 1.6), mean LA volume index (25.2 ± 5.3 mL/m^2^), and mean SPAP (23.0 ± 3.9 mmHg) did not significantly differ between males and females. As an adjunctive measure of diastolic function indicators, the resting LA strain values were examined. The absolute values for reservoir, conduit, and contraction strain were 28.1%, 14.4%, and 13.8%, respectively, with no significant difference between males and females. Generally, there was no significant change in baseline demographic and echocardiographic data between sexes, except for lower body mass index among females (Table 1).

### 3.2. Exercise-Induced Hemodynamic Changes

**Figure 2:**
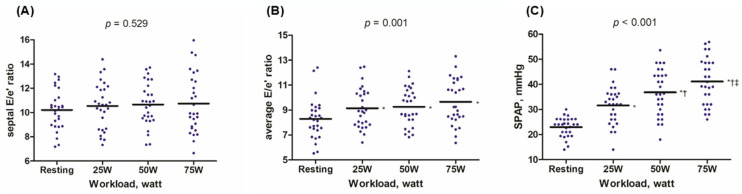
Exercise-induced hemodynamic changes. During exercise, the average E/e’ ratio and systolic pulmonary artery pressure (SPAP) gradually increased (*p* for trend < 0.05, panel (**B**,**C**)), while the septal E/e’ ratio remained stable (*p* for trend = 0.529, panel (**A**)). Although the average E/e’ ratio showed no additional increase as the exercise workload increased from 25 W to 75 W, the E/e’ ratio after starting exercise was slightly higher than the resting value (* *p* < 0.05 in each exercise workload versus resting). Regarding SPAP, a stepwise increase was observed with each workload (* *p* < 0.05 in each exercise workload compared to resting; † *p* < 0.05 compared to 25 W; ‡ *p* < 0.05 compared to 50 W). Data are presented as blue dots, and black bars represent the mean. Abbreviations: SPAP, systolic pulmonary artery pressure.

During exercise, both the E wave and e’ velocity increased, resulting in the E/e’ ratio remaining almost unchanged or slightly increased. The septal E/e’ ratio changed from 10.2 ± 1.6 at rest to 10.7 ± 2.4 at 75 W (*p* for trend = 0.529); the average E/e’ ratio changed from 8.3 ± 1.6 at rest to 9.7 ± 1.8 at 75 W (*p* for trend = 0.001). However, the peak average E/e’ ratio remained below 10 (Figure 2 and Supplementary Figure S1). SPAP gradually increased during exercise, rising from 23.0 ± 3.9 mmHg at rest to 41.2 ± 9.3 mmHg at 75 W (*p* < 0.001, Figure 2C). Additionally, we examined the dynamic changes in the RV-PA coupling index (TAPSE/SPAP, mm/mmHg) during exercise. The TAPSE/SPAP levels decreased from 0.76 ± 0.18 at rest to 0.48 ± 0.16 during exercise (Table 2). The comprehensive echocardiographic data collected throughout the entire exercise session, encompassing systolic parameters, RV functional measurements, and information on the recovery phase, are outlined in the Supplementary Table S1.

### 3.3. Exercise-Induced Hemodynamic Changes Based on Sex

**Figure 3:**
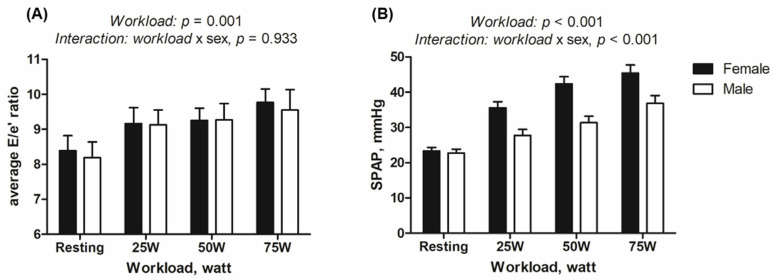
Exercise-induced hemodynamic changes based on sex. Regarding the average E/e’ ratio (panel (**A**)), the pattern of increase was similar between sexes (interaction *p* = 0.933), although females consistently exhibited numerically higher values at each workload. As for systolic pulmonary artery pressure (panel (**B**)), the pattern of increase during exercise varied between sexes (interaction *p* < 0.001), with a steeper increase observed in females. The error bars represent the standard error of the mean. Abbreviations: SPAP, systolic pulmonary artery pressure.

Overall, females demonstrated a shorter total exercise time (558.6 ± 81.7 versus 687.1 ± 69.4 s, *p* < 0.001) and a greater heart rate elevation at 75 W compared to males (137.8 ± 13.7 versus 111.4 ± 15.7 bpm at 75 W, *p* < 0.001; 68.7 ± 12.9 versus 39.3 ± 14.0 bpm for ΔHR_75W_, *p* < 0.001, Supplementary Table S2). Females showed a numerically greater E/e’ ratio during exercise, although it was not statistically significant (11.0 ± 2.5 versus 10.5 ± 2.6 for septal E/e’ at 75 W, *p* = 0.620; 9.8 ± 1.4 versus 9.6 ± 2.2 for average E/e’ at 75 W, *p* = 0.762, Table 3). Despite comparable resting SPAP, females exhibited a significantly greater SPAP elevation at 75 W (45.5 ± 8.3 versus 36.8 ± 8.3 mmHg, *p* = 0.011, Table 3). As depicted in Figure 3, the trajectory of SPAP change during exercise differed based on sex, with a steeper elevation in SPAP from the early stage of exercise (interaction *p* for workload * sex < 0.001). Females demonstrated a more depressed RV-PA coupling index at 75 W (0.41 ± 0.10 versus 0.59 ± 0.18, *p* = 0.009, Table 3).

### 3.4. Reproducibility of the Exercise Echocardiographic Parameters

We assessed the reliability of exercise echocardiographic parameters by randomly selecting 20 studies. The measurements of diastolic parameters at rest, 25 W, 50 W, and 75 W (peak exercise) were conducted by two experienced staff members (MHJ and HOJ). Inter- and intra-observer reproducibility were both excellent, with ICCs > 0.9 across all workloads (Supplementary Tables S3 and S4).

## 4. Discussion

This prospective pilot cohort study investigated the exercise hemodynamic responses of asymptomatic middle-aged to older adults using bicycle exercise echocardiography. Intriguingly, exertional dyspnea was detected in two individuals (7%), only revealed through exercise testing, leading to their exclusion from the final analyses. Our findings showed a slight elevation in the E/e’ ratio during exercise, with a mean peak value ranging from 10 to 11 and rarely exceeding 13. We observed a gradual increase in SPAP during exercise, with a mean peak value of 41 mmHg. SPAP generally remained below 50 mmHg, with none exceeding 60 mmHg. Sex-specific analysis showed a slightly higher E/e’ ratio in females during exercise, with no statistically significant difference between sexes. However, significant differences were noted in SPAP values and their patterns of increase based on sex. Females exhibited significantly elevated SPAP during exercise and a steeper SPAP increase from the early stage of exercise compared to males. Additionally, our data indicate a more pronounced depression of the RV-PA coupling index in females compared to males. Collectively, the current study provides reference exercise hemodynamic values and patterns, as well as sex-specific characteristics, in an asymptomatic population.

### 4.1. Normal Exercise Hemodynamic Profiles in Asymptomatic Older Adults

Our study aimed to establish normal reference values for diastolic parameters during exercise in asymptomatic middle-aged to older adults. The mean age of our cohort was 61 years, with 60.7% aged between 60 and 74. Notably, 61% had hypertension and 25% had diabetes; however, all had well-controlled conditions without overt cardiovascular disease or exercise-limiting symptoms, making them representative of their age group. The mean LA reservoir strain value in our cohort was 28.1 ± 6.2%, which aligns with the normal range for middle-aged to older Korean adults, based on data from a large Korean registry. In this registry, the mean LA reservoir strain was 31.2 ± 5.9% for individuals aged 50–59 and 27.7 ± 6.2% for those aged 60–69 [20]. The resting average E/e’ in our cohort was 8.3, which corresponds well with the resting diastolic parameters of the Asian middle-aged to older population (average E/e’ of 7.4 in adults aged 41–65 and 8.5 in adults aged ≥ 65) [21]. In a study involving young adults aged under 40 years (mean age of 29 years old), peak-exercise septal E/e’ and average E/e’ were 7.2 and 6.6, respectively [15]. Another study focused on middle-aged healthy adults (mean age of 59 years) who underwent treadmill exercise and reported lower resting and peak-exercise septal E/e’ values of 6.7 and 6.6, respectively, compared to our cohort’s results (10.2 and 10.7, respectively). This difference could be attributed to the fact that all participants in that cohort had no previous history of hypertension, were slightly younger than our cohort population, and peak E/e’ values were acquired after the cessation of treadmill exercise [13]. It appears that normal reference values for peak-exercise E/e’ ratio may vary according to age groups, ethnicity, and exercise protocol.

The current analyses provide additional evidence by offering comprehensive data on exercise hemodynamics, encompassing not only the E/e’ ratio but also SPAP, alongside other ancillary parameters of RV function, such as RV/PA coupling index and RV free wall strain. In the normal population, limited data exist regarding SPAP changes during exercise, let alone other RV parameters. Tracking exercise-induced changes in SPAP is crucial when performing exercise echocardiography, especially in individuals experiencing exertional dyspnea. Unlike the rapid rise in SPAP observed early in exercise for patients with HFpEF, asymptomatic individuals typically exhibit a gradual increase in SPAP, with an average peak value of 41 mmHg, usually not exceeding 50 mmHg (corresponding to a peak TR velocity of 3.0 m/s, typically not exceeding 3.4 m/s). In the literature, a TR velocity cutoff of >2.8 m/s at rest is commonly accepted for suspicion of HFpEF. However, for abnormal TR velocity during exercise, the suggested cutoff values for HFpEF diagnosis range from 2.8 m/s to 3.4 m/s, with a lack of evidence supporting a definitive threshold [7,22,23]. According to a review paper on stress echocardiography, the suggested abnormal TR velocity value is >2.8 m/s for both resting and peak exercise [7]. Although this lower cutoff during exercise could enhance HFpEF diagnosis sensitivity, it may also raise false positive rates. The 2021 European Society of Cardiology guideline suggests > 3.4 m/s peak exercise TR velocity as abnormal, but evidence supporting this is weak [22,24]. Previous studies involving healthy subjects have shown that the upper limit of normal peak TR velocity during exercise ranges from 2.8 to 3.0 m/s, consistent with our findings (converting to SPAP 36–41 mmHg) [23,25,26]. A previous study found that an invasively measured exercise SPAP ≥ 45 mmHg is useful for diagnosing HFpEF [27]. In the study, exercise SPAP was 35 mmHg in normal individuals aged 47 years and 59 mmHg in HFpEF patients aged 65 years. Additional studies across various demographics are needed to establish definitive upper normal limits and abnormal cutoff values for SPAP or TR velocity during exercise.

Additionally, we assessed several RV parameters. The TAPSE/SPAP ratio decreased gradually during exercise, from 0.72 to 0.48 mm/mmHg. Guazzi et al. previously reported that a lower TAPSE/SPAP ratio (<0.35) was associated with adverse outcomes in HFpEF [19]. Unlike most studies that only measured the TAPSE/SPAP ratio at rest, our study tracked it throughout the entire exercise period, including resting, 25 W, 50 W, peak exercise, and the recovery phase. RV free wall strain showed no significant change overall but exhibited a trend of elevation. However, RV free wall strain at 75 W appeared slightly decreased, likely due to poor tracking during tachycardia. Conversely, RV free wall strain at 2 min into recovery was higher than that at 50 W (see Supplementary Table S1). Identifying RV free wall strain at 2 min post-exercise might provide an alternative estimate of peak exercise RV free wall strain.

### 4.2. Sex-Specific Data During Exercise

The sex-specific analysis revealed a slightly higher E/e′ ratio in females during exercise, though this difference was not statistically significant. In contrast, more pronounced differences were observed in both the values and trajectories of SPAP. Females exhibited significantly higher SPAP during exercise and a steeper increase from early workload stages, along with a more marked reduction in the RV-PA coupling index. These findings partially support the well-established female predominance in HFpEF [28,29,30]. Consistent with our observations, Beale et al. reported that women with overt HFpEF demonstrated higher exercise LVFP and lower PA compliance than men [31]. In our asymptomatic adult cohort, sex differences in pulmonary vascular reserve appeared to be more prominent than those in LV diastolic reserve, as reflected by significant differences in SPAP and RV-PA coupling, but not in E/e′. Whether this discrepancy reflects differences in pathophysiological stage—our cohort representing stage A–B HFpEF and that of Beale et al., comprising overt HFpEF (stage C)—or other population-specific factors remains uncertain. The females in our study also had a numerically higher resting LVEF, a finding that may be partially attributed to sex-related differences in LV geometry, including a smaller LV chamber size [28]. These structural characteristics may limit the stroke volume reserve and necessitate greater heart rate augmentation to maintain cardiac output during exertion. In line with this, females exhibited a more rapid heart rate rise and shorter total exercise duration. These physiological patterns likely reflect the combined influence of smaller body size, heightened autonomic responsiveness, and potentially lower cardiorespiratory fitness [28,29,32].

Several biological mechanisms may underlie these sex-specific findings. Prior studies have shown that women have smaller pulmonary artery diameters, a higher proportion of small vessels, and lower pulmonary vascular compliance [31,33]. These anatomical and functional traits may amplify pressure responses to rising cardiac output, contributing to the steeper SPAP increases and greater declines in the TAPSE/SPAP ratio observed in females. These changes indicate higher RV afterload and less efficient RV-PA coupling under stress. Hormonal influences may also play a role. Estrogen is known to regulate vascular tone, remodeling, and inflammatory pathways [34]. Notably, women with higher parity (≥3 childbirths) have demonstrated greater pulmonary vascular stiffening [35], potentially due to reduced cumulative estrogen exposure. Although we did not assess menopausal status or hormone levels in our study, the influence of hormonal milieu on pulmonary vascular adaptation remains a relevant area for future research.

### 4.3. Clinical Implications

Our findings may provide useful reference values for interpreting exercise echocardiography, particularly in patients undergoing the test due to exertional dyspnea. In our cohort of asymptomatic adults, an exercise E/e′ ratio < 13 and a gradual rise in SPAP appeared to reflect a normal hemodynamic response. Conversely, if the E/e′ ratio exceeds 13—especially if it rises to ≥15—or if TR velocity exceeds 3.0 m/s (corresponding to SPAP ≥ 40 mmHg) during early exercise stages (e.g., 25 W or 50 W), such responses may indicate abnormal elevations in LVFP or pulmonary pressures. Clinically, these findings could raise suspicion for subclinical HFpEF, particularly in patients presenting with unexplained exertional dyspnea. In such cases, further evaluation, including invasive hemodynamic assessment with right heart catheterization, may be warranted.

### 4.4. Study Limitations

The present study has several limitations to consider. Firstly, the small sample size is a major limitation and precludes a detailed analysis of age group-specific data, including the impact of menopause on exercise hemodynamics in women. Although sex-specific analyses were presented, this study was not statistically powered to detect robust sex-based differences, and the related findings should be interpreted as exploratory. These important questions warrant further investigation in future studies with larger and appropriately powered cohorts. Moreover, while our inclusion criteria encompassed adults aged 45–75 years, which could be perceived as relatively broad, the actual enrolled participants had a mean age of 60.9 ± 8.4 years, resulting in a narrower distribution compared to previous reference studies, such as the Mayo Clinic study (59 ± 14 years) [13] and the Yonsei Medical Center study (38 ± 14 years) [14]. Thus, despite some degree of age-related heterogeneity remaining, we believe our study population offers a reasonable compromise for an exploratory pilot study, and we agree that future studies should aim for more age-stratified designs. In real-world practice, exercise echocardiography is typically performed in individuals with symptoms or structural heart disease. Consequently, this test is rarely conducted in asymptomatic, relatively healthy individuals, leading to a scarcity of data on exercise hemodynamics in this population. To address this gap, we collected data specifically for research purposes rather than clinical use. However, recruiting participants from an asymptomatic population proved challenging, and while we ultimately fulfilled our initial target for participants, it took nearly one year to do so. Despite the small sample size, we believe the data collected are valuable, as they provide insights into a population that is underrepresented in the current literature—namely, asymptomatic middle-aged to older adults. In particular, our study offers novel contributions by providing reference data on exercise hemodynamics in this demographic, capturing dynamic changes during early submaximal exercise and during the recovery phases at 2 and 5 min post-exercise, and incorporating additional echocardiographic markers such as LV strain, RV strain, and RV-PA coupling (Supplementary Table S1). Secondly, we did not conduct simultaneous cardiac catheterization during exercise in asymptomatic individuals, as it is an invasive procedure. Accordingly, SPAP was estimated noninvasively using TR velocity, with a fixed RAP of 5 mmHg. While this approach is commonly used in exercise echocardiography protocols, it is subject to the inherent limitations of non-invasive estimation. In particular, individual RAP may vary within a physiological range (typically 5–10 mmHg), which could affect the accuracy of SPAP calculation. In our cohort, all participants demonstrated a euvolemic profile with an inferior vena cava diameter <1.5 cm and >50% inspiratory collapse, supporting the use of a standardized RAP of 5 mmHg. Nevertheless, we acknowledge that this fixed assumption may not fully reflect individual variations and could have introduced some degree of measurement error in SPAP estimation. Thirdly, our cohort does not exclusively represent perfectly healthy adults without hypertension or diabetes. Participants with these conditions may have had some degree of ventricular or atrial structural changes, which could potentially influence exercise hemodynamics. However, only individuals with well-controlled hypertension (blood pressure < 140/90 mmHg) and well-controlled diabetes (HbA1c < 7.0%) were included. Importantly, mean blood pressure and LV mass index did not significantly differ between those with and without hypertension (Supplementary Table S5), suggesting that the influence of these comorbidities on the observed results was likely limited. Fourthly, as this study was primarily designed for descriptive analysis of exercise hemodynamics, we did not control for potential confounding factors. Unmeasured variables such as smoking, medications, other laboratory results, sleep deprivation, and stress levels may have influenced the observed exercise hemodynamics [36]. While the participants did not report significant variations in sleep quality or stress, these factors could still have affected the results. Future studies should address these variables to provide more comprehensive insights. Additionally, although TAPSE, RV strain, and LV strain were not the main parameters of interest, some missing values occurred due to technical limitations. These were considered missing at random and are unlikely to have introduced systematic bias. Lastly, this study was conducted at a single tertiary hospital, which may introduce selection bias. Nevertheless, our cohort represents asymptomatic middle-aged to older adults who may be at risk for developing HFpEF, offering valuable insights in an area where data are currently limited.

## 5. Conclusions

This exploratory pilot study investigated the exercise hemodynamic responses of asymptomatic middle-aged to older adults using bicycle exercise echocardiography. The detection of exertional dyspnea in a small percentage of individuals underscores the importance of exercise testing in uncovering subtle symptoms. Our findings provide valuable reference values for exercise-induced changes in key parameters, such as the E/e’ ratio and SPAP, in asymptomatic adults who may be at risk for future HFpEF. Furthermore, we identified sex-specific differences in hemodynamic responses, with females exhibiting a more pronounced and rapid elevation in SPAP and a greater decline in the RV/PA coupling index, findings that may help explain the female predominance of HFpEF. These observations enhance our understanding of normal exercise hemodynamics and may contribute to early identification of individuals at risk for HFpEF.

## Figures and Tables

**Table 1:** Baseline characteristics of study population.

Variable	All Patients (*n* = 28)	Female (*n* = 14)	Male (*n* = 14)	*p*
Clinical variables				
Age, y	60.9 ± 8.4	59.7 ± 8.0	62.0 ± 8.9	0.480
Systolic blood pressure, mmHg	119.3 ± 7.7	118.1 ± 7.4	120.4 ± 8.0	0.426
Diastolic blood pressure, mmHg	75.0 ± 8.5	71.7 ± 7.2	78.4 ± 8.6	0.036
Body mass index, kg/m^2^	24.6 ± 4.0	22.6 ± 4.3	26.6 ± 2.3	0.007
Hypertension, n (%)	17 (60.7)	7 (50.0)	10 (71.4)	0.440
Diabetes mellitus, n (%)	7 (25.0)	1 (7.1)	6 (42.9)	0.077
Echocardiographic variables				
LV ejection fraction, %	62.9 ± 2.3	63.3 ± 1.8	62.4 ± 2.7	0.325
IVSd, mm	9.9 ± 1.3	9.1 ± 0.9	10.8 ± 1.1	<0.001
LVMI, g/m^2^	87.1 ± 16.7	83.5 ± 18.6	90.8 ± 14.1	0.247
Septal E/e’ ratio	10.2 ± 1.6	10.5 ± 1.6	9.9 ± 1.7	0.383
Average E/e’ ratio	8.3 ± 1.6	8.4 ± 1.6	8.2 ± 1.7	0.743
SPAP, mmHg	23.0 ± 3.9	23.3 ± 3.8	22.8 ± 4.1	0.721
LA volume index, mL/m^2^	25.2 ± 5.3	24.5 ± 4.8	25.9 ± 6.0	0.490
LA strain				
LA reservoir, %	28.1 ± 6.2	28.1 ± 5.5	28.2 ± 7.0	0.953
LA conduit, %	−14.4 ± 4.5	−15.4 ± 5.2	−13.4 ± 3.7	0.250
LA contraction, %	−13.8 ± 5.9	−12.6 ± 5.2	−14.9 ± 6.5	0.314

Abbreviations: IVSd, interventricular septal thickness at end-diastole; LA, left atrium; LV, left ventricle; LVMI, left ventricular muscle index; SPAP, systolic pulmonary artery pressure. Continuous variables were compared between males and females using Student’s *t*-test, while categorical variables were analyzed using the Chi-square test or Fisher’s exact test, as appropriate.

**Table 2:** Exercise-induced hemodynamic changes in the asymptomatic population.

	Resting	25 W	50 W	75 W	*p*
Systolic BP, mmHg	140.0 ± 14.9	161.2 ± 21.0	162.9 ± 40.8	179.1 ± 41.5	<0.001
Pulse pressure, mmHg	56.6 ± 10.9	78.3 ± 20.1	81.4 ± 25.5	92.6 ± 27.8	<0.001
Heart rate, bpm	70.6 ± 10.2	91.8 ± 9.5	105.1 ± 13.2	124.6 ± 19.8	<0.001
Septal E/e’ ratio	10.2 ± 1.6	10.5 ± 1.9	10.7 ± 1.8	10.7 ± 2.4	0.529
Average E/e’ ratio	8.3 ± 1.6	9.1 ± 1.6	9.3 ± 1.5	9.7 ± 1.8	0.001
TR Vmax, m/s	2.1 ± 0.2	2.5 ± 0.4	2.8 ± 0.4	3.0 ± 0.4	<0.001
SPAP, mmHg	23.0 ± 3.9	31.6 ± 7.5	36.9 ± 9.1	41.2 ± 9.3	<0.001
TAPSE/SPAP *	0.76 ± 0.18	0.62 ± 0.20	0.54 ± 0.18	0.48 ± 0.16	<0.001

Abbreviations: BP, systolic blood pressure; SPAP, systolic pulmonary artery pressure; TAPSE, tricuspid annular plane systolic excursion. * The availability of TAPSE/SPAP varied during different stages of the exercise due to unreliable TAPSE measurements. Specifically, TAPSE measurements were unreliable for certain patients during specific exercise stages, leading to fluctuations in the availability of TAPSE/SPAP ratios: 24 individuals at rest, 23 at 25 W, 21 at 50 W, and 20 at 75 W, respectively. The summarized TAPSE/SPAP data represent results from 20 individuals for whom both TAPSE and SPAP could be adequately measured throughout the entire exercise stage.

**Table 3:** Sex-specific echocardiographic profiles.

	Female (*n* = 14)	Male (*n* = 14)	*p*
Resting profiles			
Systolic blood pressure, mmHg	140.4 ± 18.0	139.6 ± 11.7	0.882
Pulse pressure, mmHg	59.8 ± 10.0	53.4 ± 11.2	0.126
Heart rate, bpm	69.1 ± 10.4	72.1 ± 10.0	0.434
Septal E/e’ ratio	10.5 ± 1.6	9.9 ± 1.7	0.383
Average E/e’ ratio	8.4 ± 1.6	8.2 ± 1.7	0.743
SPAP, mmHg	23.3 ± 3.8	22.8 ± 4.1	0.721
TAPSE/SPAP *, mm/Hg	0.74 ± 0.12	0.82 ± 0.29	0.389
75 W exercise profiles			
Systolic BP, mmHg	192.6 ± 22.0	165.6 ± 51.9	0.085
Pulse pressure, mmHg	106.3 ± 21.5	78.9 ± 27.1	0.006
Heart rate, bpm	137.8 ± 13.7	111.4 ± 15.7	<0.001
Septal E/e’ ratio	11.0 ± 2.5	10.5 ± 2.5	0.620
Average E/e’ ratio	9.8 ± 1.4	9.6 ± 2.2	0.762
SPAP, mmHg	45.5 ± 8.3	36.8 ± 8.3	0.011
TAPSE/SPAP *, mm/Hg	0.41 ± 0.10	0.59 ± 0.18	0.009

Abbreviations: SPAP, systolic pulmonary artery pressure; TAPSE, tricuspid annular plane systolic excursion. * TAPSE measurements were available for 24 individuals at rest (13 females and 11 males), and for 20 individuals at peak exercise (75 W) (12 females and 8 males). Otherwise, all data in the table above were available for all 28 individuals.

## Data Availability

All relevant data are included in this manuscript.

## Supplementary Materials

The following supporting information can be downloaded at: https://www.mdpi.com/article/10.3390/diagnostics15111307/s1, Table S1: Exercise-induced hemodynamic changes throughout the entire exercise period; Table S2: Exercise-induced changes; Table S3: Intra-observer intraclass correlation coefficients (ICCs); Table S4: Inter-observer intraclass correlation coefficients (ICCs); Table S5: hemodynamic and structural cardiac parameters in participants with and without a history of hypertension; Figure S1: Violin plots of exercise-induced changes at each workload.

## Abbreviations

The following abbreviations are used in this manuscript:

HFpEF	Heart failure with preserved ejection fraction
ICC	Intraclass correlation coefficient
LA	Left atrial
LV	Left ventricular
LVEF	Left ventricular ejection fraction
LVFP	Left ventricular filling pressure
PA	Pulmonary artery
rpm	Rotations per minute
RV	Right ventricle
SPAP	Systolic pulmonary artery pressure
TAPSE	Tricuspid annular plane systolic excursion
