# Supplementary material for: Exercise Hemodynamics and Sex-Specific Data in Asymptomatic Adults: An Exploratory Pilot Study
Source: Diagnostics (Basel). 2025 May 23;15(11):1307. doi: 10.3390/diagnostics15111307 (PMC12155339; doi:10.3390/diagnostics15111307)
Supplement: Supplementary file 1 [file diagnostics-15-01307-s001.zip › diagnostics-3564221-supplementary.pdf]

Table S1. Exercise-induced Hemodynamic Changes Throughout the Entire Exercise Period

	Resting	25W	50W	75W	Rec2min	Rec5min	*P*
Heart rate, bpm	70.6 ± 10.2	91.8 ± 9.5	105.1 ± 13.2	124.6 ± 19.8	95.9 ± 13.8	86.0 ± 10.7	<0.001
Systolic BP, mmHg	140.0 ± 14.9	161.2 ± 21.0	162.9 ± 40.8	179.1 ± 41.5	158.6 ± 18.3	140.5 ± 15.8	<0.001
Diastolic BP, mmHg	83.4 ± 9.5	82.9 ± 19.2	81.5 ± 32.8	86.5 ± 27.0	82.6 ± 11.6	80.1 ± 10.6	0.612
Pulse pressure, mmHg	56.6 ± 10.9	78.3 ± 20.1	81.4 ± 25.5	92.6 ± 27.8	76.0 ± 17.4	60.4 ± 10.8	<0.001
E velocity, cm/s	61.1 ± 12.1	90.0 ± 15.3	101.4 ± 21.3	115.2 ± 22.4	71.6 ± 17.3	65.9 ± 15.2	<0.001
A velocity, cm/s	61.9 ± 12.3	87.5 ± 19.0	95.0 ± 25.9	111.7 ± 28.7	85.8 ± 22.0	73.8 ± 19.6	<0.001
Septal e’ velocity, cm/s	6.1 ± 1.1	8.7 ± 1.4	9.6 ± 2.0	11.1 ± 2.9	7.5 ± 1.5	6.8 ± 1.3	<0.001
Lateral e’ velocity, cm/s	8.9 ± 1.9	11.3 ± 2.5	12.6 ± 3.0	13.3 ± 3.3	10.1 ± 1.9	9.7 ± 2.1	<0.001
Average e’ velocity, cm/s	7.5 ± 1.4	10.0 ± 1.7	11.1 ± 2.4	12.2 ± 2.8	8.8 ± 1.6	8.2 ± 1.6	<0.001
Septal E/e’ ratio	10.2 ± 1.6	10.5 ± 1.9	10.7 ± 1.8	10.7 ± 2.4	9.7 ± 2.2	9.9 ± 2.0	0.042
Average E/e’ ratio	8.3 ± 1.6	9.1 ± 1.6	9.3 ± 1.5	9.7 ± 1.8	8.3 ± 2.0	8.1 ± 1.9	<0.001
TR Vmax, m/s	2.1 ± 0.2	2.5 ± 0.4	2.8 ± 0.4	3.0 ± 0.4	2.4 ± 0.3	2.2 ± 0.3	<0.001
SPAP, mmHg	23.0 ± 3.9	31.6 ± 7.5	36.9 ± 9.1	41.2 ± 9.3	29.9 ± 6.4	26.0 ± 4.7	<0.001
TAPSE/SPAP*	0.72 ± 0.11	0.59 ± 0.16	0.51 ± 0.16	0.48 ± 0.16	0.63 ± 0.18	0.66 ± 0.14	<0.001

RVFWS, %†	24.1 ± 4.4	24.9 ± 5.5	25.0 ± 5.4	23.2 ± 4.2	26.1 ± 5.9	24.4 ± 5.5	0.060
LVEF, %	62.9 ± 2.3	63.9 ± 2.5	65.8 ± 2.3	66.9 ± 2.8	67.2 ± 4.0	64.9 ± 2.5	<0.001
LVGLS, %‡	16.9 ± 1.3	17.9 ± 1.7	18.4 ± 1.9	17.2 ± 7.0	18.7 ± 2.5	18.0 ± 2.2	0.304

Abbreviations: BP, systolic blood pressure; LVEF, left ventricle ejection fraction; LVGLS, left ventricle global longitudinal strain; SPAP, systolic pulmonary artery pressure; Rec2min, post-exercise recovery 2 minute; Rec5min, post-exercise recovery 5 minute; RVFWS, right ventricle free wall strain; TAPSE, tricuspid annular plane systolic excursion; TR, tricuspid regurgitation.

The availability of certain parameters (TAPSE/SPAP, RVFWS, and LVGLS) varied across different stages of the exercise due to unreliable measurements. Specifically, TAPSE measurements was unreliable for some patients during certain exercise stages, resulting in varying availability of TAPSE/SPAP ratios: 24 individuals at resting, 23 at 25W, 21 at 50W, 20 at 75W, 21 at Rec2min, and 24 at Rec5min, respectively. Summarized data represent results from 18 individuals for whom both TAPSE and SPAP could be adequately measured throughout the entire exercise stage. This principle, ensuring complete echocardiographic measurements during the whole exercise period, was similarly applied to RVFWS and LVGLS.

*TAPSE/SPAP ratio was available for 18 individuals.

†RVFWS was available for 20 individuals.

‡LVGLS was available for 22 individuals

Table S2. Exercise-induced Changes

	Overall	Female (n=14)	Male (n=14)	*P*
**Changes between 25W and Resting (Δ_{25W})**				
Δ_{25W} Septal E/e' ratio	0.37 ± 2.17	0.45 ± 2.07	0.29 ± 2.33	0.849
Δ_{25W} Average E/e' ratio	0.85 ± 1.49	0.77 ± 1.13	0.93 ± 1.82	0.784
Δ_{25W} SPAP, mmHg	8.6 ± 5.7	12.3 ± 4.4	5.0 ± 4.5	<0.001
Δ_{25W} RVFWS, %	1.3 ± 4.2	2.4 ± 4.7	-0.2 ± 3.2	0.142
Δ_{25W} LVEF, %	1.0 ± 1.2	1.1 ± 1.3	0.8 ± 1.1	0.474
Δ_{25W} LVGLS, %	1.0 ± 1.4	1.2 ± 1.7	0.8 ± 1.1	0.489
Δ_{25W} Heart rate, bpm	21.1 ± 9.6	25.6 ± 10.6	16.6 ± 6.0	0.010
Δ_{25W} TAPSE/SPAP, mm/mmHg	0.14 ± 0.24	0.18 ± 0.05	0.08 ± 0.30	0.359
**Changes between 50W and Resting (Δ_{50W})**				
Δ_{50W} Septal E/e' ratio	0.42 ± 2.04	0.40 ± 1.99	0.43 ± 2.17	0.974
Δ_{50W} Average E/e' ratio	0.97 ± 1.58	0.86 ± 1.67	1.07 ± 1.54	0.736
Δ_{50W} SPAP, mmHg	13.8 ± 7.7	19.1 ± 5.4	8.6 ± 5.9	<0.001
Δ_{50W} RVFWS, %	0.7 ± 4.6	1.2 ± 5.5	-0.1 ± 3.1	0.501

Δ_{50W} LVEF, %	2.9 ± 1.8	3.4 ± 2.2	2.4 ± 1.3	0.178
Δ_{50W} LVGLS, %	1.5 ± 1.8	1.9 ± 1.5	1.2 ± 2.1	0.348
Δ_{50W} Heart rate, bpm	34.5 ± 13.6	43.1 ± 13.0	25.9 ± 7.5	<0.001
Δ_{50W} TAPSE/SPAP, mm/mmHg	0.21 ± 0.21	0.28 ± 0.16	0.10 ± 0.25	0.048
**Changes between 75W and Resting (Δ_{75W})**				
Δ_{75W} Septal E/e’ ratio	0.60 ± 2.49	0.49 ± 2.4	0.71 ± 2.7	0.814
Δ_{75W} Average E/e’ ratio	1.37 ± 2.19	1.37 ± 2.15	1.36 ± 2.31	0.984
Δ_{75W} SPAP, mmHg	18.1 ± 8.3	22.2 ± 6.7	14.1 ± 7.9	0.007
Δ_{75W} RVFWS, %	-0.9 ± 3.5	-1.63 ± 3.51	0.07 ± 3.39	0.282
Δ_{75W} LVEF, %	4.0 ± 2.5	4.3 ± 3.0	3.7 ± 2.1	0.535
Δ_{75W} LVGLS, %	0.32 ± 7.3	-2.22 ± 9.03	3.07 ± 3.35	0.067
Δ_{75W} Heart rate, bpm	54.0 ± 20.0	68.7 ± 12.9	39.3 ± 14.0	<0.001
Δ_{75W} TAPSE/SPAP, mm/mmHg	0.27 ± 0.24	0.33 ± 0.12	0.19 ± 0.35	0.295

Abbreviations: LVEF, left ventricle ejection fraction; LVGLS, left ventricle global longitudinal strain; SPAP, systolic pulmonary artery pressure; right ventricle free wall strain; TAPSE, tricuspid annular plane systolic excursion.

Changes in RVFWS, LVGLS, and TAPSE/SPAP ratio were available for some participants. TAPSE/SPAP ratio data were available for 28, 23, 21, and 20 individuals during resting, 25W, 50W, and 75W stages, respectively. RVFWS data were available for 25, 23, 24, and 24 individuals at each stage, while LVGLS data were available for 25 individuals throughout the exercise stages.

Table S3. Intra-observer intraclass correlation coefficients (ICCs)

Parameter	Rest	25W	50W	75W
Septal E/e′ ratio	0.987	0.983	0.950	0.973
Average E/e′ ratio	0.991	0.981	0.957	0.968
SPAP, mmHg	0.981	0.924	0.991	0.913

Abbreviations: SPAP, systolic pulmonary artery pressure.

Table S4. Inter-observer intraclass correlation coefficients (ICCs)

**Parameter**	**Rest**	**25W**	**50W**	**75W**
Septal E/e′ ratio	0.968	0.953	0.932	0.953
Average E/e′ ratio	0.987	0.976	0.957	0.957
SPAP, mmHg	0.981	0.941	0.990	0.978

Abbreviations: SPAP, systolic pulmonary artery pressure.

Table S5. Hemodynamic and structural cardiac parameters in participants with and without a history of hypertension

	No history of hypertension (n=11)	History of hypertension (n=17)	***P***
Systolic BP, mmHg	118.2 ± 7.8	119.9 ± 7.8	0.563
Diastolic BP, mmHg	74.5 ± 6.4	75.4 ± 9.8	0.777
IVSd, mmHg	9.6 ± 1.5	10.1 ± 1.2	0.314
LVMI, g/m^2	88.5 ± 19.9	86.3 ± 14.8	0.742

BP, blood pressure; IVSd, interventricular septal thickness at end-diastole; LVMI, left ventricular mass index.

Figure S1. Violin plots of exercise-induced changes at each workload

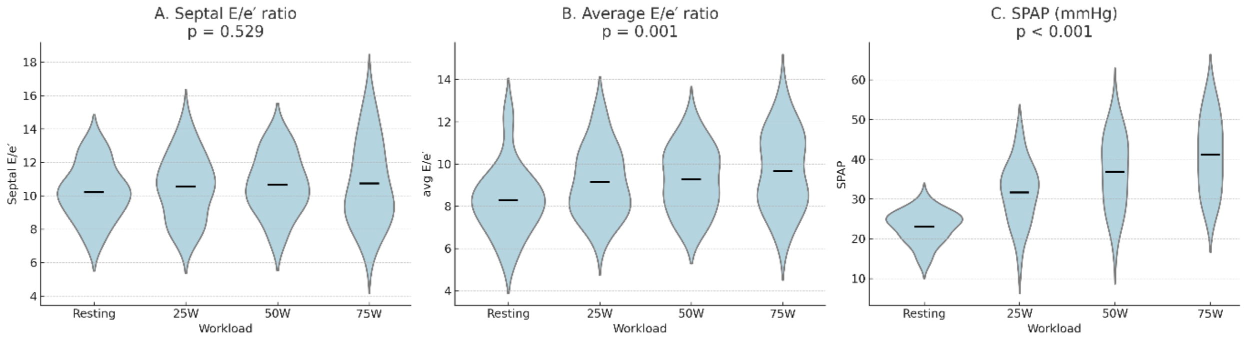

Abbreviations: SPAP, systolic pulmonary artery pressure.

Violin plots illustrating the distributions of septal E/e′ ratio, average E/e′ ratio, and systolic pulmonary artery pressure (SPAP) at four levels of exercise workload (resting, 25W, 50W, and 75W). Black bars indicate the mean values at each workload. Violin plots represent the data distribution, including density and variability. These plots complement the main Figure 2 by providing a more comprehensive view of the data distribution, including variability and skewness.
